# The Impact of Intermittent Hypoxic Training on Aerobic Capacity and Biometric-Structural Indicators among Obese Women—A Pilot Study

**DOI:** 10.3390/jcm13020380

**Published:** 2024-01-10

**Authors:** Małgorzata Bagińska, Anna Kałuża, Łukasz Tota, Anna Piotrowska, Marcin Maciejczyk, Dariusz Mucha, Ibrahim Ouergui, Rafał Kubacki, Olga Czerwińska-Ledwig, Dorota Ambroży, Kazimierz Witkowski, Tomasz Pałka

**Affiliations:** 1 Institute of Biomedical Sciences, Department of Physiology and Biochemistry, University of Physical Education in Kraków, 31-571 Kraków, Polandtomasz.palka@awf.krakow.pl (T.P.); 2 Department of Chemistry and Biochemistry, Faculty of Physiotherapy, University of Physical Education in Krakow, 31-571 Kraków, Poland; 3 Department of Body Renovation and Body Posture Correction, Faculty of Physical Education and Sport, University of Physical Education in Kraków, 31-571 Kraków, Poland; 4 Sports Science, Health and Movement, High Institute of Sport and Physical Education of Kef, University of Jendouba, El Kef 7100, Tunisia; 5 Faculty of Physical Education and Sports, Wroclaw University of Health and Sport Sciences, 51-612 Wroclaw, Poland; 6 Institute of Sports Sciences, University of Physical Education in Krakow, 31-571 Kraków, Poland; 7 Faculty of Physical Education and Sports, University of Physical Education in Wrocław, 31-571 Kraków, Poland

**Keywords:** normobaric hypoxia, training in hypoxia, obesity, physiological parameters

## Abstract

Background: Obesity, a common lifestyle-related condition, is correlated with factors like inadequate physical activity. Its connection to diverse health issues presents a significant challenge to healthcare. This pilot study investigated the effects of hypoxic training on aerobic capacity and biometric-structural indicators in obese women. The secondary objective was to determine the feasibility, effectiveness, and safety of the planned research procedures and their potential for larger-scale implementation. Material and methods: Forty-one non-trained women with first-degree obesity were randomly assigned to even normobaric hypoxic training (H + E), normoxic training (E), passive exposure to hypoxia (H), and a control group (C). Training sessions were conducted three times a week for four weeks (12 training sessions). Body composition parameters were assessed, metabolic thresholds were determined, and maximal oxygen consumption (VO_2_max) was measured before and after interventions. Results: The results demonstrated that training in hypoxic conditions significantly affected somatic parameters, with the H + E group achieving the best outcomes in terms of weight reduction and improvements in body composition indicators (*p* < 0.001). Normoxic training also induced a positive impact on body weight and body composition, although the results were less significant compared to the H + E group (*p* < 0.001). Additionally, training in hypoxic conditions significantly improved the aerobic capacity among the participants (*p* < 0.001). The H + E group achieved the best results in enhancing respiratory endurance and oxygen consumption (*p* < 0.001). Conclusions: The results of this pilot study suggest, that hypoxic training can be effective for weight reduction and improving the aerobic capacity in obese women. Despite study limitations, these findings indicate that hypoxic training could be an innovative approach to address obesity and related conditions. Caution is advised in interpreting the results, considering both the strengths and limitations of the pilot study. Before proceeding to a larger-scale study, the main study should be expanded, including aspects such as dietary control, monitoring physical activity, and biochemical blood analysis.

## 1. Introduction

Obesity, which is one of the most common health problems occurring in modern society, represents a complex medical condition with its etiology linked to various factors. One of the key risk factors for the development of obesity is a lack of regular physical activity. Due to reduced physical activity, the body accumulates excess fat tissue, leading to metabolic disturbances and other severe health complications [1]. Obesity is not only an aesthetic concern but, more importantly, increases significantly the risk of type 2 diabetes and cardiovascular diseases [1]. Excessive body mass is ranked as the sixth most important risk factor responsible for the number of deaths worldwide [2,3,4,5]. According to the World Health Organization (WHO), obesity is considered as a global epidemic, making it one of the most important non-communicable chronic diseases [6]. In the context of clinical and epidemiological significance, research is being conducted on the pathogenesis of obesity and its coexisting conditions. Recent findings highlight the significance of the inflammatory process, resulting from the increased metabolic activity of adipocytes, in the development of both obesity and its associated diseases [7]. Excessive fat tissue accumulation adversely affects overall health and quality of life. This is related to the increase in the volume of adipocytes, which affects the secretion of adipokines (reduced adiponectin production and increased leptin secretion) [8].

Hypoxic training has become a popular approach in the sports community, attracting the attention of both athletes and researchers seeking effective methods to improve performance at sea level [9]. The combination between physical training and hypoxia exposure has also been proposed as a new therapeutic strategy aiming to improve the overall health in overweight and obese individuals [10]. The increasing prevalence of obesity and its coexisting diseases not only imposes a significant health burden, but also carries significant economic consequences, creating serious challenges for healthcare systems and generating treatment costs [6]. Overweight and obesity affect nearly 60% of the European population and are responsible for more than 1.2 million deaths annually (13% of all deaths). In Europe, obesity is slightly more common among women (25%) than men (24%) [6]. Therefore, there is a valid reason for searching new strategies to facilitate effective weight loss and the treatment of coexisting diseases in obese individuals.

Positive adaptations of the body due to regular physical activity are widely documented in the literature [11,12]. Physical activity significantly influences body composition, corrects posture defects, increases lung capacity, and demonstrates a decrease in one’s resting heart rate and a reduction in blood pressure values during physical exertion [13]. Physical exercises strengthen the immune system, and their regular practice has a positive impact on overall health, serving as therapeutic support and preventing or aiding in the treatment of many conditions [14]. With age and the development of industrialization, a decline in physical activity is observed, accompanied by a series of involutionary changes. A lack of physical activity contributes to health deterioration and predisposes individuals to various lifestyle-related diseases, including obesity [15].

Training conducted under hypoxic conditions has an impact on increasing the activity of vascular endothelial growth factor (VEGF), which leads to the expansion of the blood vessel network [16], and results in the better oxygenation of adipose tissue, thereby intensifying lipolysis processes and reducing fat tissue [17,18]. In hypoxic conditions, appetite is also reduced due to the increased release of cholecystokinin and leptin, as well as a decrease in ghrelin levels [19]. Recent research findings indicate that training sessions conducted in hypoxic conditions contribute to increased fat tissue reduction and lower blood cholesterol levels [17,18,20], which has favorable implications for the treatment of obesity and related diseases. It is also shown that the training in hypoxic conditions is safe for the skin—it positively affects stratum corneum hydration, without significantly altering the function of the skin barrier or its firmness [21].

The development and evaluation of the effectiveness of a training program conducted under normobaric hypoxic conditions appear to be a promising approach in the context of obesity prevention and the reduction of its negative consequences. There are potential practical implications to consider in terms of effective training, improving lipid metabolism regulation, reducing inflammation, and enhancing physical performance.

Therefore, the aim of this pilot study was to investigate the impact of intermittent hypoxic training on aerobic capacity and biometric-structural indicators among obese women. The secondary objective was to determine the feasibility, effectiveness, and safety of the planned research procedures and their potential for larger-scale implementation. Additionally, this study aimed to establish optimal training loads and environmental conditions (normobaric hypoxia) while also enhancing the clinical experience of the research team with the described intervention. We hypothesized that incorporating hypoxia into training would promote greater fat reduction while significantly improving the aerobic capacity of obese women.

## 2. Materials and Methods

### 2.1. Participants Characteristics

Forty-one non-training women of (age: 41.3 ± 10.5 years), who were diagnosed with first-degree obesity, according to the World Health Organization (WHO) standards [17], volunteered to participate in this pilot study. The inclusion and exclusion criteria for/from this study are presented in Table 1.

**Figure 1:**
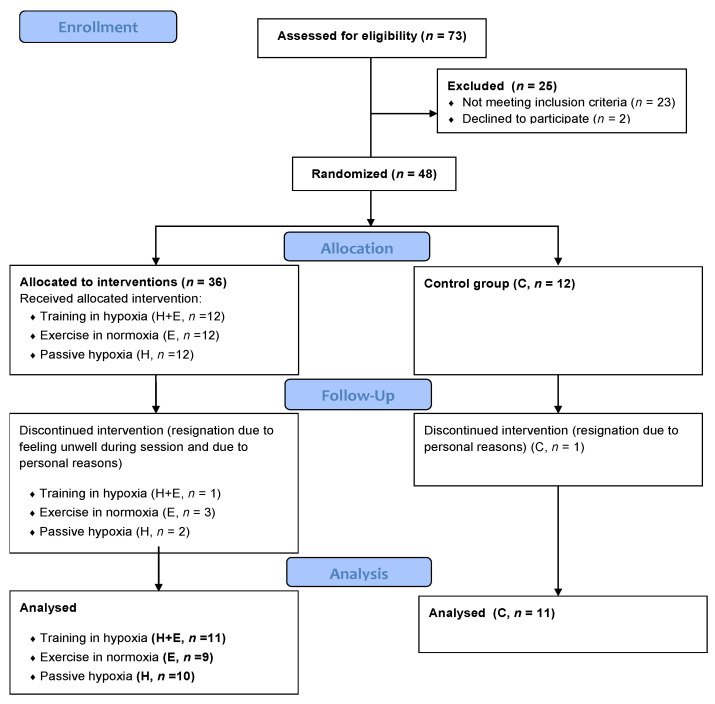
CONSORT 2010 Flow Diagram.

The participants were randomly assigned to one of four groups: training in normobaric hypoxia (H + E), training in normoxia (E), passive exposure to hypoxia (H), and a control group without any intervention (C) (Figure 1). During the study, the women did not change their dietary habits and did not use any substances, vitamins, or other supplements.

The project obtained approval from the Ethics Committee at the District Medical Chamber in Krakow, under the reference number 70/KBL/OIL/2022. The participants were informed about the purpose and course of the research and provided written consent to participate. In accordance with the Helsinki Declaration requirements, the participants were informed about the research objectives, methods, potential side effects, and the option to withdraw from the study at any time without providing a reason. The research was conducted under the supervision of qualified medical personnel and took place at the Central Laboratory for Scientific Research of the University of Physical Education in Krakow, following PN-EN ISO 9001:2015 standards. Prior to the study, the participants underwent medical qualification, where they were found to have no contraindications to physical activity.

### 2.2. Study Protocol

**Figure 2:**
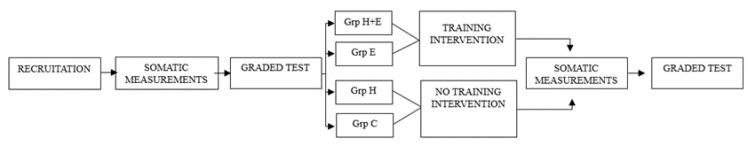
Study protocol.

Before starting the training intervention, basic anthropometric measurements and essential exercise tests evaluating the aerobic capacity of the participants were conducted. Training loads were determined based on a graded exercise test, and a 4-week training cycle was initiated. Approximately 7–10 days after its completion, anthropometric and physiological assessments were repeated (Figure 2).

### 2.3. Somatic Measurements

Somatic measurements, including body composition and body mass (BM), were determined using the JAWON MEDICAL IOI-353 body composition analyzer (Jawon Medical CO, Seoul, Republic of Korea), taking into account the requirements of MDD Directive 93/42EEC for medical devices. The requirements were taken into account: Current Status of Body Composition Assessment in Sport [22]. To assess body composition, an eight-electrode bioelectrical impedance technique was employed to determine lean body mass (LBM), percentage of body fat (%BF), and fat mass (FM). Height (BH) was measured using stadiometer (Seca, Hamburg, Germany) with a measurement accuracy of 0.5 cm. The Body Mass Index (BMI), a weight-to-height ratio, was calculated based on BM and BH.

### 2.4. Graded Exercise Test

A direct method was employed to assess aerobic capacity, involving a graded exercise test conducted on a cycle ergometer (ER 900 D—72475 BIT2 by Jeager, Friedberg, Germany) until the participant reached exhaustion due to extreme fatigue. During the test, physiological indicators were determined at the second ventilatory threshold (VT2) and at the maximal oxygen consumption (VO_2_max) level. To determine VT2, changes in respiratory indicators were analyzed as exercise intensity increased. The criteria for determination of VT2 were as follows: (a) the percentage of CO_2_ in exhaled air reached its maximum value and then decreased, (b) the respiratory exchange ratio for carbon dioxide reached its minimum value and then increased, and (c) a non-linear, significant increase in lung ventilation was observed after crossing VT2. VO_2_max was defined as the highest recorded value.

The exercise test was preceded by a three-minute warm-up during which the participant pedaled at a frequency of 60 revolutions per minute with power out of 90 W. Then, every 2 min, the power output was increased by 20 W. The test was performed until the participant refused to continue due to extreme fatigue.

During the test, the following indicators were recorded using an ergospirometer (Cortex Metalyzer 3B, Leipzig, Germany): pulmonary ventilation (VE), oxygen consumption per minute (VO_2_), carbon dioxide production per minute (VCO_2_), and the respiratory exchange ratio (RER). Heart rate (HR) during the test was measured using a sport tester (H7, Polar, Kempele, Finland).

All physiological tests were conducted in a climate-controlled laboratory in the morning hours, no sooner than 2 h after a light meal.

### 2.5. Training Program

Women in the H + E group trained under conditions simulating an altitude of 2500 m above sea level at an FiO_2_ (fraction of inspired oxygen) of 15.4% and a temperature of 21 °C. Participants in the E group performed the same training in normoxia (200 m above sea level, temperature 21 °C). Training sessions were conducted three times a week for a period of four weeks, totaling 12 training sessions. The workloads for the training were individually determined on the basis of a graded test performed in normoxia: the power output corresponding to 85% HRmax and 70% HRmax was determined. Interval training was performed on a Monark cycle ergometer type 834 E (Varberg, Sweden) according to the following scheme: 6 min of exercise with power out corresponding to 85%HRmax and 6 min active recovery at power output corresponding to 70% HRmax (i.e., 12 min × 5 repetitions = 60 min). Women in the H group were passively exposed to hypoxic conditions (2500 m above sea level, FiO_2_ = 15.4%, temperature 21 °C), while participants in the C group did not undergo any intervention.

The training sessions took place in a hypoxic thermoclimatic chamber (Hypoxico, Bickenbach, Germany), where ambient temperature and relative air humidity were controlled using a Harvia thermo-hygrometer (Harvia, Muurame, Finland) and an Ellab electrothermometer (Ellab, Hillerød, Denmark) with measurement accuracy of ±3% and ±0.5 °C. Air movement was monitored using a Hill cathermometer (Hill-Rom GmbH, Essen, Germany).

To ensure the highest level of precaution among the study participants, who constitute a specific group (obese women) during the training program, a medical consultation was conducted, individualization of loads was applied, warm-up and cool-down exercises were implemented, heart rate and oxygen saturation were monitored during exercises, and the entire process was supervised by medical personnel.

### 2.6. Statistical Analysis

The sample size was determined priori using G * Power version 3.1.9.2 (Dusseldorf, Germany), and the following parameters were assumed as a statistical test: f-tests family and ANOVA with repeated measures (4 groups); the statistical power was 0.9, the significance level was 0.01, and the effect size was 0.4. The power analysis indicated that a minimum sample size of 40 women was required for the pilot study.

The statistical analyses were performed using IBM SPSS Statistics version 28 software package (IBM Inc., Chicago, IL, USA). The Shapiro–Wilk W test was used to verify the normality of distributions. With intention of verifying the effects of the type of intervention on the changes in the abovementioned indicators, a mixed ANOVA was conducted: Condition (Control vs. Hypoxia vs. Hypoxia and Exercise vs. Exercise) × Series (First Measurement vs. Second Measurement). The pairwise comparisons were calculated with the Bonferroni correction. In case of BMI in Hypoxia and Exercise Condition, additionally, the Wilcoxon Signed Rank Test for paired samples was applied (due to non-normal distribution). Additionally, the effect size was determined. Since mixed ANOVA was used, partial eta squared (η^2^*_p_*) was considered for the effect size (following the interpretation: 0.01—indicates the small effect, 0.06—medium effect, 0.14—large effect). An alpha level of 0.05 was used for all analyses.

## 3. Results

### 3.1. Somatic Parameters

For the Hypoxia and Exercise and Exercise groups, the BMI significantly lowered in the second measurement compared to the first (Table 2 and Table 3). No differences in the percentage of body fat were observed in the hypoxia group after observation. However, in the hypoxia and exercise and exercise groups, lower values of BF were recorded after the training (*p* < 0.001, *p* = 0.003). LBM significantly increased after the training in all three experimental groups (Table 2 and Table 3). Regarding the control group, no significant differences were observed between the first and second measurements in all the somatic indicators. In all three groups (hypoxia, hypoxia and exercise, exercise), a significant decrease in body mass, the body mass index, and fat mass was recorded in the second measurement in comparison to the baseline (Table 3).

### 3.2. Metabolic Thresholds

Concerning the three experimental conditions (i.e., Hypoxia, Hypoxia and Exercise, and Exercise), a significant increase in power output at the second ventilatory threshold was recorded. A significant increase in the relative oxygen consumption was observed only in women in the Hypoxia and Exercise group. An increase in exercise intensity at VT2 expressed as %HR was observed only in the Hypoxia and Exercise group. No changes in the level of any of the investigated indicators were observed in the control group (Table 2 and Table 4).

### 3.3. Aerobic Capacity

In the control group, no significant changes were observed regarding the absolute and relative VO_2max_ between the first and second measurements. In the three other experimental conditions, the maximal workload and relative oxygen uptake significantly increased after the interventions. A significant increase in the absolute VO_2_max was only recorded in women from the Hypoxia and Exercise group; no difference was noted in the other groups. Maximal pulmonary ventilation significantly increased in two groups, the Hypoxia and Exercise and Exercise groups, while this improvement was not observed in the Hypoxia group. No significant changes in the maximal heart rate were observed in any of the investigated conditions (Table 2 and Table 5).

## 4. Discussion

Hypoxia is often perceived as a pathological state or a symptom of disease [23,24]; however, it can also serve as a therapeutic tool in the treatment of obesity and its coexisting conditions, such as cardiovascular, respiratory, and nervous system diseases [25]. Compensatory positive mechanisms and the body’s responses to hypoxia have been presented, involving the respiratory system (improved respiratory function and increased diffusion in lungs), the cardiovascular system (elevated heart rate, peripheral vasodilation, and blood pressure normalization), cellular and metabolic processes (angiogenesis, increased insulin sensitivity, enzymatic activation, and mitochondrial biogenesis), as well as body mass regulation (leptin modulation, activation of the adrenergic system, appetite suppression, and serotonin production) [25,26]. Hypoxia, when applied under controlled conditions, in obesity and its coexisting diseases, can be a safe alternative to traditional weight control approaches such as dietary interventions, exercise programs, pharmacology, or surgical procedures [27].

It is commonly known that obesity results from a disturbed energy balance caused by excessive energy intake, limited physical activity, or a combination of both factors [28]. In the context of obesity treatment, achieving a negative energy balance, where energy expenditure exceeds energy intake, is crucial for weight management [29]. It has long been known that hypoxia affects the energy balance and can lead to weight loss. However, to date, most reports have only focused on field conditions by managing factors such as changes in dietary habits, activity patterns, and environmental conditions that could influence weight loss [30]. In our present research, when participants were exposed to isolated hypoxic conditions, a significant decrease in BM, BMI, and FM was observed. A previous study has also reported weight loss and increased energy expenditure in obese individuals submitted to passive hypoxia [31]. Weight loss in response to hypoxic conditions’ exposure may be associated with the activation of hypoxia-inducible factor-1 (HIF-1) [32]. Both passive and active hypoxia stimulate the production of HIF-1, which is associated with improved glucose metabolism, oxygen transport, and satiety, thereby reducing the risk of obesity and normalizing body weight [31]. It is also worth noting that a hypoxia stimulus itself led to an increase in lean body mass while reducing BM, indicating a reduction in fat tissue while preserving muscle mass. Muscles are metabolically active and consume more oxygen compared to fat tissue, so maintaining muscle mass at a high level is important for preserving metabolic efficiency and overall physical fitness [33]. These data suggest the possibility of a direct impact of hypoxic conditions on the regulation of body weight and composition.

It is widely known that oxygen is essential for fat burning. In obese individuals, adipose tissue is reported to be poorly oxygenated, contributing to chronic inflammation and the development of obesity-related diseases [27]. Importantly, all metabolic processes in adipose tissue depend on blood flow [34]. Exposure to hypoxic conditions influences the activity of endothelial growth factor, leading to an increased capillary density and an improved blood, including oxygen, flow to the adipose tissue [12]. Additionally, engaging in physical activity in hypoxia increases the number and efficiency of muscle mitochondria and oxidative enzymes [25]. Being in conditions of hypoxia also enhances lipolysis through the activation of adipose protein kinase [35] and improves the glucose uptake in human adipocytes by expressing glucose transporter genes and GLUT-1 protein [36]. As a result of these mechanisms induced by hypoxic conditions, the more efficient oxidation of adipose tissue and a reduction in fat mass can be expected upon returning to normoxic conditions.

Increasing one’s physical activity level is likely the most important action in preventing and treating obesity [37]. In the presented study, interval training conducted under normoxic conditions had a positive impact on the body mass and composition of the participants, leading to a significant decrease in the BM, BMI, and FM while increasing the LBM. However, compared to the normoxic counterpart, training under hypoxic conditions resulted in a greater percentage decrease in the BMI, BM, FM, and LBM increase in the study group. Additionally, only the H + E group showed a statistically significant decrease in PF, which was not observed in the other groups. A meta-analysis conducted by Zhijian He et al. [38] demonstrated a significantly greater decrease in the BM and FM indices in the hypoxic training groups compared to normoxic conditions. In the mentioned meta-analysis, LBM also showed a significant improvement in the hypoxic groups, but this effect was not significantly higher than equivalent normoxic interventions [38]. However, Gatter et al. [39] reported that, among 32 obese individuals, no significant difference in weight loss was recorded between hypoxic and normoxic training interventions. Similarly, no positive results from previous studies were achieved [40] in terms of body weight management in response to hypoxic training. Therefore, there are conflicting pieces of evidence regarding the effectiveness of training in hypoxic conditions as a tool for improving weight loss. Differences in the results obtained may be explained by the use of different training protocols, including varying intensity, intervention duration, and the percentage of hypoxia in which the training took place. Based on the present study’s results and those of previous research, we can only observe a tendency in which exercises in moderate hypoxia (FiO_2_ > 15%) [38,41] induced a greater impact on improving the body mass index than more severe hypoxia (FiO_2_ ≤ 15%) [39,40,42]. However, this requires further researchers using consistent training protocols at different altitudes. In summary, both passive exposure to hypoxic conditions and training under normoxic and hypoxic conditions have a positive impact on the body composition profile and body mass of participants. Training under hypoxic conditions appears to be a stronger factor influencing weight loss and body composition improvement than training under normoxic conditions. However, significant discrepancies in the training protocols used in the available literature suggest the need for further research to find optimal training patterns.

Training in hypoxic conditions is a factor that has a positive effect on cardiorespiratory fitness among athletes, which has already been confirmed by numerous researchers [43,44]. However, despite the growing interest in hypoxic conditions as a therapeutic method in the treatment of obesity and related diseases, there is still no scientific consensus about the impact of hypoxic training on cardiorespiratory indicators in overweight individuals [45]. In our own study, training under hypoxic conditions significantly improved women’s aerobic capacity for both relative and absolute values. It is worth noting that the other groups showed also a significant improvement in oxygen consumption, although this increase was not much pronounced compared to the H + E group. Camacho-Cardenosa et al. [46] demonstrated that, within 82 obese women, interval hypoxic training (IHT) and repeated sprint hypoxic training (RSH) significantly increased absolute VO_2_max values (IHT: +26.63%; RSH: +19.79%) and relative VO_2_max (IHT: +27.95%; RSH: +19.94%) between the baseline and post-exercise values compared to groups performing the same training under normoxic conditions. The aerobic capacity improvement was also confirmed by other researchers who compared high-intensity exercise protocols in hypoxia and normoxia [46,47,48]. It is worth noting that the intensity of the exercises used can also be a decisive factor influencing the results, as no additional improvement in cardiorespiratory parameters was observed when combining hypoxic conditions with low-intensity training [39,49,50]. These results suggest that high-intensity interval training may induce significant favorable physiological adaptations in a shorter time than continuous low-intensity training, and hypoxic conditions provide an additional increase in the aerobic capacity compared to normoxic training. Since the lack of time often hinders regular physical activity [51], high-intensity training in hypoxic conditions can be a time-effective strategy that encourages adherence to physical activity recommendations. However, caution should be exercised given the health status of the participants. This is particularly important for obese individuals for whom weight reduction and metabolic disease prevention are a priority.

The effectiveness of substrate utilization and maximum oxygen consumption is closely related to the oxidative capacity of muscle mitochondria [52]. Additionally, an improvement in the aerobic capacity can be correlated with quantitative and qualitative adaptations of mitochondria [53]. In hypoxic conditions, the body triggers adaptive reactions aimed at maintaining the oxygen and energy supply in the absence of sufficient oxygen [19]. Even short-term exposure to hypoxia stimulates and activates immediate and long-term mechanisms related to glycolysis, glycogenesis, and lactate transport [54,55]. Therefore, training in hypoxic conditions can contribute to reversing oxidative and mitochondrial dysfunction in obese individuals [56], thereby improving cardiorespiratory fitness and ultimately reducing the risk of cardiovascular disorders. Interestingly, in the present research, passive exposure to hypoxic conditions resulted in a greater improvement in VO_2_ compared to exercise in normoxic conditions. Previous studies have shown that cardiorespiratory fitness is inversely proportional to the development of metabolic syndrome [57], and the physical fitness level is strongly negatively correlated with the incidence of cardiovascular diseases and the mortality rate [58,59,60]. The changes observed in the response to the passive exposure to hypoxic conditions may thus represent a significant turning point in the prevention and treatment of metabolic diseases and cardiovascular dysfunction, especially in obese individuals. Hypoxia can lead to an increase in metabolic stress (relative workload) without the intensification of mechanical stress [61], which opens up new therapeutic possibilities for individuals for whom physical activity may be limited due to orthopedic issues associated with an excessive musculoskeletal load.

The power output in training corresponded to 85%HRmax and 70%HRmax (exercise and recovery, respectively), and was determined individually in a graded test. The training program (power output), was the same for each group, but training in hypoxia resulted in a different physiological response, resulting precisely from hypoxia, which was the aim of this study. Our assumption was that in each training group, the training was the same (training was not an additional variable), and the only additional factor affecting (or not) the physiological response was the environmental factor (normoxia or hypoxia).

### Strength and Limitation of the Pilot Study

The study was performed under laboratory conditions using normobaric hypoxia conditions and a controlled environment (temperature, humidity, air movement). Other environmental conditions, other than those used in this study, may induce different effects. Hypoxic conditions also occur naturally (hypobaric hypoxia), and can also be induced with the use of hypoxicators (administering a mixture of hypoxic air through a breathing mask). In our study, training took place in a hypoxic chamber, where hypoxia has a systemic effect. A different method of hypoxic training may produce different effects than those reported in this study. The use of a hypoxic chamber in this type of study is also a limitation, as it only allows for the simultaneous training of only a few participants.

The testing equipment and procedures used proved their suitability for this type of experiment. These were previously validated procedures and devices. The submaximal-intensity training used proved to be effective and well-planned in testing the hypothesis and achieving the goals assumed. Other types of training require the separate verification of effectiveness in obese individuals. The main study should be expanded to include dietary control, the monitoring of natural physical activity, further biochemical analysis, and the psychological care of the participants. Despite informing participants of the need to maintain their current dietary and physical activity habits, we cannot be sure that these recommendations were actually followed. Future studies need to be expanded to include hematological and biochemical markers to better understand the body’s adaptations to hypoxic conditions in the context of weight reduction and cardiorespiratory fitness in the obese population. The study also revealed problems related to recruiting female participants and maintaining motivation to complete the entire training cycle. Before embarking on a larger-scale study, it is necessary to identify ways to ensure that participants are comfortable with the training and appropriately motivated to complete the study.

Although the sample size in this study was calculated, the number of participants in each group was relatively small and should be considered a minimum sample size. Therefore, the results presented should be interpreted with caution and confirmed in a larger population. From preliminary calculations, the sample size in the main study could be about twice as large (76 women) under the assumptions: the statistical power 0.95, the significance level 0.05, and the effect size 0.25 (G * power, the same calculation method as described in the method section).

This pilot study shows that a research team of at least a dozen people, with clearly divided roles and responsibilities, is required to carry out this type of study. Our research team was experienced in implementing this type of study, so we noted no logistical, organizational, or communication problems. For inexperienced researchers, the prior development of communication and accountability is required.

The participants did not report any side effects that could affect the ethical evaluation of the research performed.

There is undoubtedly a need for further research to determine the optimal combination of training intensity, volume, and degree of hypoxia required to induce beneficial adaptations in the context of weight loss and improved cardiorespiratory parameters in obese women.

## 5. Conclusions

Both training in normoxia and training in hypoxia were effective in reducing body mass in a small sample size. Both groups showed an increase in the relative VO_2_max, but in the normoxia group, the increase was due to a reduction in body mass—only training in hypoxia resulted in a significant increase in the absolute VO_2_max, indicating an improvement in the aerobic capacity of this group. A greater reduction in body fat and body mass was observed in the group training in hypoxia than in the group training in normoxia. This may indicate, that exercise in hypoxia may not only be more effective in improving aerobic capacity, but also in reducing body fat, but this needs to be confirmed in a larger study population. The conclusions drawn suggest that hypoxic training could be an innovative approach to addressing obesity and related conditions. However, we recommend interpreting the presented results with caution, considering both the strengths and limitations of the conducted pilot study. Before proceeding to a larger-scale study, it is essential to consider the control of dietary habits, physical activity monitoring, and psychological care among the participants. Additionally, the study should be expanded to include biochemical and hematological markers in the blood for a better understanding of the body’s response to hypoxic training and its associated effects on weight reduction and physical fitness. Due to the complex nature of the research, an experienced research team and access to validated procedures and devices are required.

## Figures and Tables

**Table 1:** Inclusion and exclusion criteria.

**Inclusion Criteria**	**Exclusion Criteria**
→ Female gender	→ Smoking
→ Age 24–60	→ Alcohol and substance abuse
→ Medical certificate confirming no health contraindications for physical exertion	→ Dietary changes during the experiment
→ Non-use of pharmacotherapy and supplements during the study period and at least 4 weeks preceding the study	→ History of high-altitude illness in the medical history
→ Lack of regular physical activity	
→ First-degree obesity according to WHO standards (BMI = 30.0–34.9 kg/m^2^)	
→ Informed consent of the patient to participate in the study	

**Table 2:** Descriptive statistics on the variables.

Condition		Indicator	Baseline			After Training		
			M	SD	95% CI	M	SD	95% CI
Control	Morphology	BH [m]	1.66	0.07	[1.60, 1.71]	-	-	-
		BM [kg]	86.63	13.13	[77.24, 96.02]	87.25	13.94	[77.27, 97.23]
		BMI [kg/m^2^]	31.50	4.03	[28.62, 34.38]	31.71	4.18	[28.71, 34.70]
		BF [%]	38.74	3.26	[36.41, 41.07]	39.13	3.66	[36.51, 41.75]
		FM [kg]	34.11	7.64	[28.64, 39.58]	34.71	8.26	[28.80, 40.62]
		LBM [kg]	52.80	6.37	[48.24, 57.36]	52.91	6.33	[48.38, 57.43]
	Metabolic thresholds	WL VT2 [W]	127.63	29.15	[106.78, 148.49]	128.13	28.56	[107.70, 148.57]
		VO_2_ VT2 (mL/kg)	19.73	4.57	[16.46, 22.99]	19.74	4.61	[16.44, 23.03]
		%HRmax	92.74	6.12	[88.36, 97.11]	92.69	6.13	[88.31, 97.08]
	Aerobic capacity	WL [W]	153.70	47.03	[120.05, 187.35]	155.60	45.13	[123.32, 187.88]
		VEmax [L/min]	73.67	27.85	[53.75, 93.59]	75.07	25.28	[56.99, 93.15]
		HRmax (bpm)	163.00	25.89	[144.48, 181.52]	163.00	25.89	[144.48, 181.52]
		VO_2_max [mL/min]	1813.60	759.64	[1270.19, 2357.01]	1990.10	487.97	[1641.02, 2339.18]
		VO_2_max [m/kg]	22.67	4.72	[19.30, 26.04]	22.84	4.59	[19.55, 26.12]
Hypoxia	Morphology	BH [m]	1.68	0.06	[1.64, 1.72]	-	-	-
		BM [kg]	83.07	10.78	[75.36, 90.78]	81.63	11.05	[73.73, 89.53]
		BMI [kg/m^2^]	29.50	3.45	[27.03, 31.97]	29.00	3.59	[26.42, 31.57]
		BF [%]	36.66	4.24	[33.63, 39.69]	35.80	4.63	[32.49, 39.11]
		FM [kg]	30.78	6.96	[25.80, 35.75]	29.58	7.11	[24.49, 34.66]
		LBM [kg]	52.26	6.37	[48.24, 57.36]	53.16	4.96	[49.62, 56.71]
	Metabolic thresholds	WL VT2 [W]	161.00	24.78	[143.27, 178.83]	170.00	19.18	[156.28, 183.72]
		VO_2_ VT2 (mL/kg)	23.64	1.70	[22.42, 24.86]	25.47	4.27	[22.41, 28.52]
		%HRmax	94.80	1.68	[93.60, 96.00]	93.48	2.83	[91.46, 95.50]
	Aerobic capacity	WL [W]	176.50	22.46	[160.43, 192.57]	185.40	24.43	[167.93, 202.87]
		VEmax [L/min]	90.05	16.38	[78.33, 101.77]	94.08	11.56	[85.81, 102.35]
		HRmax (bpm)	181.40	15.93	[170.00, 192.80]	185.10	9.05	[178.63, 191.57]
		VO_2_max [mL/min]	2275.00	414.60	[1978.41, 2571.59]	2453.90	330.26	[2217.65, 2690.15]
		VO_2_max [mL/kg]	27.35	2.90	[25.28, 29.43]	30.14	2.08	[28.65, 31.63]
Hypoxia and Exercise	Morphology	BH [m]	1.68	0.07	[1.63, 1.72]	-	-	-
		BM [kg]	91.75	19.43	[78.69, 104.80]	86.59	17.67	[74.72, 98.46]
		BMI [kg/m^2^]	32.58	6.40	[28.28, 36.88]	30.75	5.78	[26.87, 34.63]
		BF [%]	39.92	6.57	[35.50, 44.33]	36.60	3.99	[33.92, 39.28]
		FM [kg]	40.65	18.20	[28.42, 52.87]	37.67	16.58	[26.53, 48.81]
		LBM [kg]	53.85	6.33	[49.59, 58.10]	55.87	6.40	[51.57, 60.17]
	Metabolic thresholds	WL VT2 [W]	137.27	26.46	[119.50, 155.05]	151.55	33.76	[128.86, 174.23]
		VO_2_ VT2 (mL/kg)	19.18	5.53	[14.47, 22.90]	22.36	6.15	[18.23, 26.50]
		%HRmax	95.18	2.93	[93.22, 97.15]	92.45	3.14	[90.34, 94.57]
	Aerobic capacity	WL [W]	155.18	36.83	[130.44, 179.92]	168.18	34.89	[144.74, 191.62]
		VEmax [L/min]	73.45	21.60	[58.93, 87.96]	83.00	19.37	[69.99, 96.01]
		HRmax (bpm)	156.82	23.25	[141.20, 172.44]	155.36	22.53	[140.23, 170.50]
		VO_2_max [mL/min]	1918.18	449.26	[1616.36, 2220.00]	2277.27	418.14	[1996.36, 2558.18]
		VO_2_max [mL/kg]	21.41	5.76	[17.54, 25.28]	25.45	5.82	[21.54, 29.36]
Exercise	Morphology	BH [m]	1.66	0.04	[1.63, 1.69]	-	-	-
		BM [kg]	83.47	5.56	[79.19, 87.74]	79.69	5.38	[75.56, 83.82]
		BMI [kg/m^2^]	30.38	2.01	[28.83, 31.92]	29.00	2.01	[27.46, 30.55]
		BF [%]	35.68	3.68	[32.85, 38.50]	33.57	2.85	[31.37, 35.76]
		FM [kg]	27.49	6.05	[22.84, 32.14]	25.32	5.44	[21.14, 29.50]
		LBM [kg]	48.73	4.70	[45.12, 52.35]	50.22	4.56	[46.72, 53.72]
	Metabolic thresholds	WL VT2 [W]	131.33	32.24	[106.55, 156.11]	141.00	32.19	[116.26, 165.74]
		VO_2_ VT2 (mL/kg)	22.11	5.35	[18.00, 26.22]	23.89	3.79	[20.98, 26.80]
		%HRmax	92.44	6.78	[87.23, 97.66]	91.11	6.17	[86.37, 95.86]
	Aerobic capacity	WL [W]	169.22	17.02	[156.13, 182.13]	180.67	17.48	[167.23, 194.10]
		VEmax [L/min]	79.61	17.43	[66.21, 93.01]	86.13	13.44	[75.80, 96.47]
		HRmax (bpm)	174.22	13.45	[163.88, 184.56]	174.89	13.11	[164.81, 184.97]
		VO_2_max [mL/min]	1882.22	397.17	[1576.93, 2187.51]	2047.78	395.40	[1743.84, 2351.71]
		VO_2_max [mL/kg]	22.52	4.11	[19.36, 25.68]	25.70	4.50	[22.24, 29.16]

BM—Body Mass; BMI—Body Mass Index; BF—Percentage of Body Fat; FM—Fat Mass; LBM—Lean Body Mass; WL VT2—Workload at Ventilatory Threshold 2; VO_2_ VT2—Oxygen Consumption at Ventilatory Threshold 2; %HRmax—Percentage of Maximal Heart Rate; WL—Workload; VEmax—Maximal Pulmonary Ventilation; HRmax—Maximal Heart Rate; VO_2_max [mL/min]—Maximal Oxygen Uptake; VO_2_max [mL/kg]—Relative Maximal Oxygen Uptake; M—Mean; SD—Standard Deviation; 95% CI—95% Confidence Interval.

**Table 3:** Results of mixed ANOVA and pairwise comparisons with respect to the somatic indicators.

Indicator	Mean Square	F	(df)	*p*	η^2^*_p_*	Condition	Baseline vs. after Intrvention
BM [kg]	33.414	18.70	(3.37)	<0.001	0.603	Control (*n* = 11)	*d* = −0.473, *SE* = 0.570, *p* = 0.412
						Hypoxia (*n* = 10)	*d* = 1.440, *SE* = 0.598, *p* = 0.021
						Hypoxia and Exercise (*n* = 11)	*d* = 5.155, *SE* = 0.570, *p* < 0.001
						Exercise (*n* = 9)	*d* = 3.778, *SE* = 0.630, *p* < 0.001
BMI [kg/m^2^]	4.189	18.615	(3. 37)	<0.001	0.601	Control (*n* = 11)	*d* = −0.151, *SE* = 0.202, *p* = 0.460
						Hypoxia (*n* = 10)	*d* = 0.509, *SE* = 0.212, *p* = 0.022
						Hypoxia and Exercise (*n* = 11)	*d* = 1.828, *SE* = 0.202, *p* < 0.001
						Exercise (*n* = 9)	*d* = 1.372, *SE* = 0.224, *p* < 0.001
BF [%]	13.006	6.740	(3. 37)	<0.001	0.353	Control (*n* = 11)	*d* = −0.264, *SE* = 0.592, *p* = 0.659
						Hypoxia (*n* = 10)	*d* = 0.860, *SE* = 0.621, *p* = 0.175
						Hypoxia and Exercise (*n* = 11)	*d* = 3.318, *SE* = 0.592, *p* < 0.001
						Exercise (*n* = 9)	*d* = 2.111, *SE* = 0.655, *p* = 0.003
FM [kg]	12.132	9.454	(3. 36)	<0.001	0.441	Control (*n* = 11)	*d* = −0.600, *SE* = 0.507, *p* = 0.244
						Hypoxia (*n* = 10)	*d* = 1.197, *SE* = 0.507, *p* = 0.024
						Hypoxia and Exercise (*n* = 11)	*d* = 2.973, *SE* = 0.483, *p* < 0.001
						Exercise (*n* = 9)	*d* = 2.167, *SE* = 0.534, *p* < 0.001
LBM [kg]	3.373	9.977	(3. 37)	<0.001	0.447	Control (*n* = 11)	*d* = −0.191, *SE* = 0.248, *p* = 0.446
						Hypoxia (*n* = 10)	*d* = −0.900, *SE* = 0.260, *p* = 0.001
						Hypoxia and Exercise (*n* = 11)	*d* = −2.027, *SE* = 0.248, *p* < 0.001
						Exercise (*n* = 9)	*d* = −1.489, *SE* = 0.274, *p* < 0.001

BM—Body Mass; BMI—Body Mass Index; BF—Percentage of Body Fat; FM—Fat Mass; LBM—Lean Body Mass; F—F-statistic; (df)—Degrees of Freedom; *p*—*p*-value; η^2^*_p_*—Partial Eta-squared; *d*—effect size; SE—Standard Error.

**Table 4:** Results of mixed ANOVA and pairwise comparisons at second ventilatory threshold.

Indicator	Mean Square	F	(df)	*p*	η^2^*_p_*	Condition	Baseline vs. after Intrvention
WL VT2 [W]	181.857	2.356	(3.37)	0.088	0.160	Control (*n* = 11)	*d* = −0.455, SE = 3.746, *p* = 0.904
						Hypoxia (*n* = 10)	*d* = −9.000, SE = 3.929, *p* = 0.028
						Hypoxia and Exercise (*n* = 11)	*d* = −14.273, SE = 3.746, *p* < 0.001
						Exercise (*n* = 9)	*d* = −9.667, SE = 4.141, *p* = 0.025
VO_2_ VT2 (ml/kg)	8.899	1.988	(3.37)	0.133	0.139	Control (*n* = 11)	*d* = −0.075, SE = 0.902, *p* = 0.934
						Hypoxia (*n* = 10)	*d* = −1. 827, SE = 0.946, *p* = 0.061
						Hypoxia and Exercise (*n* = 11)	*d* = −3.182, SE = 0.902, *p* = 0.001
						Exercise (*n* = 9)	*d* = −1.778, SE = 0.997, *p* = 0.083
% HRmax	3.001	0.345	(3.37)	0.793	0.027	Control (*n* = 11)	*d* = 1.106, SE = 1.258, *p* = 0.385
						Hypoxia (*n* = 10)	*d* = 1.320, SE = 1.319, *p* = 0.323
						Hypoxia and Exercise (*n* = 11)	*d* = 2.727, SE = 1.258, *p* = 0.037
						Exercise (*n* = 9)	*d* = 1.333, SE = 1.390, *p* = 0.344

WL VT2—Workload at Ventilatory Threshold 2; VO_2_ VT2—Oxygen Consumption at Ventilatory Threshold 2; %HRmax—Percentage Maximal Heart Rate; F—F-statistic; (df)—Degrees of Freedom; *p*—*p*-value; η^2^*_p_*—Partial Eta-squared; *d*—effect size; SE—Standard Error.

**Table 5:** Results of mixed ANOVA and pairwise comparisons at maximal intensity.

Indicator	Mean Square	F	(df)	*p*	η^2^*_p_*	Condition	Baseline vs. after Intrvention
WL [W]	134.368	2.869	(3.37)	=0.049	0.189	Control (*n* = 11)	*d* = −1.727, SE = 2.918, *p* = 0.557
						Hypoxia (*n* = 10)	*d* = −8.900, SE = 3.060, *p* = 0.006
						Hypoxia and Exercise (*n* = 11)	*d* = −13.000, SE = 2.918, *p* < 0.001
						Exercise (*n* = 9)	*d* = −11.444, SE = 3.226, *p* = 0.001
VEmax [L/min]	67.846	2.322	(3.37)	=0.091	0.158	Control (*n* = 11)	*d* = −1.273, SE = 2.305, *p* = 0.584
						Hypoxia (*n* = 10)	*d* = −4.030, SE = 2.417, *p* = 0.104
						Hypoxia and Exercise (*n* = 11)	*d* = −9.555, SE = 2.305, *p* < 0.001
						Exercise (*n* = 9)	*d* = −6.522, SE = 2.548, *p* = 0.015
HRmax [bpm]	31.942	1.185	(3.37)	=0.329	0.088	Control (*n* = 11)	*d* = 1.636, SE = 2.214, *p* = 0.465
						Hypoxia (*n* = 10)	*d* = −3.700, SE = 2.322, *p* = 0.120
						Hypoxia and Exercise (*n* = 11)	*d* = 1.455, SE = 2.214, *p* = 0.515
						Exercise (*n* = 9)	*d* = −0.667, SE = 2.448, *p* = 0.787
VO_2_max [mL/min]	48347.6	1.151	(3.37)	=0.341	0.085	Control (*n* = 11)	*d* = −164.64, SE = 87.38, *p* = 0.067
						Hypoxia (*n* = 10)	*d* = −178.90, SE = 91.65, *p* = 0.059
						Hypoxia and Exercise (*n* = 11)	*d* = −359.09, SE = 87.38, *p* < 0.001
						Exercise (*n* = 9)	*d* = −165.56, SE = 96.61, *p* = 0.095
VO_2_max [mL/kg]	14.977	11.316	(3.37)	<0.001	0.478	Control (*n* = 11)	*d* = −0.194, SE = 0.491, *p* = 0.695
						Hypoxia (*n* = 10)	*d* = −2.790, SE = 0.514, *p* < 0.001
						Hypoxia and Exercise (*n* = 11)	*d* = −4.046, SE = 0.491, *p* < 0.001
						Exercise (*n* = 9)	*d* = −3.184, SE = 0.542, *p* < 0.001

WL—Workload; VEmax—Maximal Pulomonary Ventilation; HRmax—Maximal Heart Rate; VO_2_max [mL/min]—Maximal Oxygen Uptake; VO_2_max [mL/kg]—Relative Maximal Oxygen Uptake; F—F-statistic; (df)—Degrees of Freedom; *p*—*p*-value; η^2^*_p_*—Partial Eta-squared; *d*—effect size; SE—Standard Error.

## Data Availability

All data are included in the manuscript.
